# Current Insights into Oral Cancer Epigenetics

**DOI:** 10.3390/ijms19030670

**Published:** 2018-02-27

**Authors:** Alexandra Iulia Irimie, Cristina Ciocan, Diana Gulei, Nikolay Mehterov, Atanas G. Atanasov, Diana Dudea, Ioana Berindan-Neagoe

**Affiliations:** 1 Department of Prosthetic Dentistry and Dental Materials, Division Dental Propaedeutics, Aesthetics, Faculty of Dentistry, “Iuliu Hatieganu” University of Medicine and Pharmacy, 400001 Cluj-Napoca, Romania; iulia.irimie@umfcluj.ro (A.I.I.); ddudea@umfcluj.ro (D.D.); 2 MEDFUTURE—Research Center for Advanced Medicine, “Iuliu Hatieganu” University of Medicine and Pharmacy, Marinescu 23 Street, 400001 Cluj-Napoca, Romania; crisciocan@yahoo.com (C.C.); diana.c.gulei@gmail.com (D.G.); 3 Department of Medical Biology, Faculty of Medicine, Medical University-Plovdiv, 15-А Vassil Aprilov Blvd., 4000 Plovdiv, Bulgaria; ni_ki82@abv.bg; 4 Technological Center for Emergency Medicine, 15-АVassil Aprilov Blvd., 4000 Plovdiv, Bulgaria; 5 Institute of Genetics and Animal Breeding of the Polish Academy of Sciences, 05-552 Jastrzebiec, Poland; atanas.atanasov@univie.ac.at; 6 Department of Pharmacognosy, University of Vienna, Althanstrasse 14, A-1090 Vienna, Austria; 7 Research Center for Functional Genomics, Biomedicine and Translational Medicine, “Iuliu Hatieganu” University of Medicine and Pharmacy, Marinescu 23 Street, 400337 Cluj-Napoca, Romania; 8 Department of Functional Genomics and Experimental Pathology, The Oncology Institute “Prof. Dr. Ion Chiricuţă”, 400015 Cluj-Napoca, Romania

**Keywords:** oral cancer, epigenetics, DNA methylation, histone, miRNA, non-coding RNAs

## Abstract

Epigenetic modifications have emerged into one of the cancer hallmarks, replacing the concept of malignant pathologies as being solely genetic-based conditions. The epigenetic landscape is responsible for normal development but also for the heterogeneity among tissues in terms of gene expression patterns. Dysregulation in these mechanisms has been associated with disease stage, and increased attention is now granted to cancer in order to take advantage of these modifications in terms of novel therapeutic strategies or diagnosis/prognosis tools. Oral cancer has also been subjected to epigenetic analysis with numerous studies revealing that the development and progression of this malignancy are partially induced by an altered epigenetic substrate together with genetic alterations and prolonged exposure to environmental risk factors. The present review summarizes the most important epigenetic modifications associated with oral cancer and also their potential to be used as new therapeutic targets.

## 1. Introduction

Cancer stands among the most complex human diseases with multiple levels of regulation. Its complexity and heterogeneity sustain the development and advancement of carcinogenesis. Despite the significant progress that has been made in fundamental and clinical research, the incidence and mortality rates associated with malignant pathologies are still at high levels with an expected increased rate in the next 15 years [1]. Head and neck cancers rank as the sixth most common malignancy, where oral cancer represents the most frequent subtype within this spectrum [2,3]. Moreover, oral squamous cell carcinoma (OSCC) accounts for approximately 90% of the oral subtype, being by far the most common type of malignancy within the oral cavity [4,5].

The induction and development of oral cancer are due to a sum of genetic changes combined with environmental risk factors (especially tobacco and alcohol consumption but also viral infections and chronic inflammations) that in the end lead to alterations in the activity of oncogenes and tumor suppressor genes [6,7]. The level of complexity behind the abnormal expression and function of these genes is extremely high, where epigenetic mechanisms are standing as another segment of regulation besides the actual changes in the DNA sequence, which include mutations, deletions, and amplifications [8]. While epigenetic mechanisms are essential for sustaining the development and tissue-specific homeostasis of the organism, a deregulation in these processes can lead to the installation of pathological states, and increased attention is being granted nowadays toward cancer [9]. Therefore, the classical concept where cancer is controlled mainly by genetic modifications has now shifted toward a more comprehensive picture where DNA methylation, modifications of histones, and nucleosome positioning are now considered to play a crucial role. Also, the expression of non-coding RNAs (ncRNAs), especially microRNAs (miRNAs), may be influenced and at the same time is also influencing the epigenetic mechanisms [10].

Along with other types of cancers, oral malignancies have also been subjected to intense research in the last years regarding the specific modifications within the epigenome. The goal has been to obtain a more detailed profile of this disease and also to possibly develop novel diagnostic, prognostic, and therapeutic tools. The present review aims to present the most important aspects regarding the epigenetic changes that have been found until now in oral cancers and how these modifications can be used in the patient’s favor.

## 2. Epigenetics—An Emerging Concept

The basic characterization of the epigenetic concept states that these mechanisms are reversible changes that are not related to modifications in the structure of DNA and can be inherited and preserved for multiple generations. Increased research has extended the initial characterization made in 1942 by C. H. Waddington: “the causal interactions between genes and their products, which bring the phenotype into being.” The definition was primarily referring to embryonic development—“the study of heritable changes in gene expression that occur independent of changes in the primary DNA sequence,”—a characterization that is further applicable to the whole organism and stages of development, including disease states [9]. Actually, epigenetic modifications enable the possibility of heterogeneity between cells despite the same genetic material and similar cellular signaling patterns.

Research advances in the study of cancer complexity have revealed that the epigenetic landscape is different between malignant and normal cells, a fact that influences the progression of the disease and is also involved in all stages of malignant development [11,12]. Nevertheless, the reality that these non-genetic modifications are reversible is opening the possibility of new therapeutic strategies, where targeted agents can be used to restore what is considered the normal conduit in cell signaling and impair cancer development [13].

**Figure 1:**
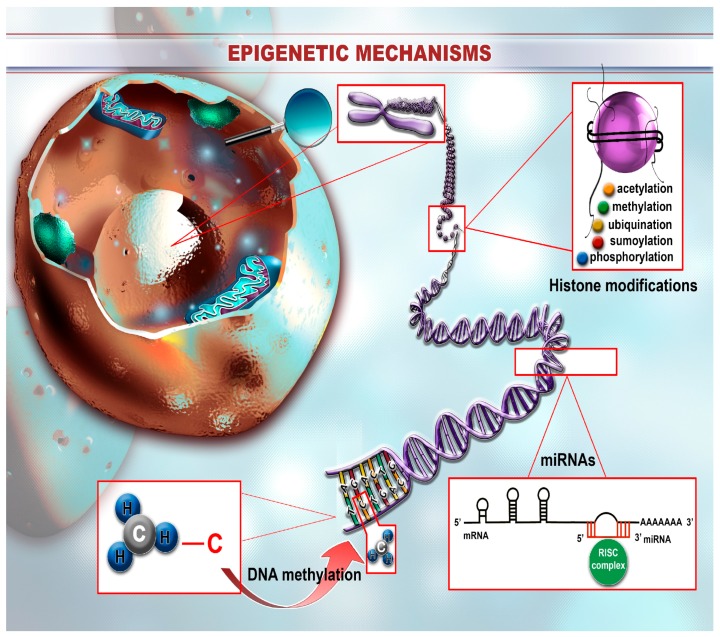
The landscape of epigenetic mechanisms. Epigenetic changes consist of reversible modifications affecting the structure of the DNA/chromosome that are further translated at the protein level through expressional changes. One of the most studied epigenetic mechanisms consists of DNA methylation that occurs mainly within the CpG islands that are located in different repetitive genome regions or, more often, within promoter regions. The methylation pattern is different depending on the region, where most of the islands located in the promoter area are hypomethylated, the other ones, from repetitive segments, are methylated. Histone modifications are also part of the epigenetic machinery and mainly consist of ubiquitylation, sumoylation, methylation, acetylation, and also phosphorylation of the histone tails. Depending on the type of modification, these mechanisms can result in increased activity of the specific DNA segment or inversely blockage of function. Not in the least, miRNAs also play a crucial part in the establishment of the epigenetic landscape where these sequences are differentially expressed between different cellular entities and also between normal and pathological cells. Their ability to target and inhibit the translation of specific genes makes them crucial players within homeostatic signaling pathways and also therapeutic targets in disease states.

The structure of chromatin consists of nucleosome units that in turn are composed of DNA wrapped around eight histone proteins [14]. This structure can be modified through epigenetic mechanisms that are mainly divided into four sectors: DNA methylation, covalent modifications of histones, non-covalent modifications—such as the introduction of histone variants and remodeling of nucleosome structure—and non-coding RNA-related modifications, especially miRNAs (Figure 1). These mechanisms act together to create the specific epigenetic landscape associated with a certain population of cells or a process within the organism. Modifications in the epigenetic signature can contribute to the induction of abnormal cellular signaling where cells lose their initial identity and become pathogenic. This is the case of malignant diseases where, besides the complex genetic background, epigenetic processes are adding an additional level of complexity in terms of cancer regulatory mechanisms.

## 3. DNA Methylation

Within the main mechanisms responsible for epigenetic regulation, DNA methylation is by far the most studied, being responsible for gene silencing and chromatin architecture. These processes take place in concordance with other epigenetic modifications in order to ensure the specific regulatory landscape and to control the phenotype of the target cell. DNA methylation primarily materializes within the so-called “CpG islands” that are composed of a series of CpG dinucleotide structures located usually at the 5′ end of genes. The CpG dinucleotides can also be found in genome regions that contain repetitive sequences, such as in the case of retrotransposon elements, rDNA, and centromeric repeats [15,16,17]. While most of the CpG sites within repetitive sequences are methylated, in the case of the grouped ones (CpG islands) that usually occupy more than half of the promoter of a certain gene, the pattern is consistently unmethylated for both undifferentiated and differentiated tissues [18]. There are also some isolated cases where CpG islands are methylated, as in the case of the X-chromosome that is inactivated during development and also for some genes associated with incipient development stages that are silenced through methylation in adult tissues [15,17,19]. For the case of dispersed CpG islands, the heavily methylated status is used to impair chromosomal instability by inhibiting the expression of transposable elements and also non-coding sequences [18].

Global analysis of CpG status in terms of methylation marks has proven to be useful for profiling the cancer epigenetic landscape. Therefore, it has been shown that approximately 5–10% of the CpG islands are aberrantly methylated within malignant pathologies, a fact that leads to the silencing of specific coding and non-coding genes (e.g., tumor suppressor genes) and implicit propagation of altered signals within specific pathways [20]. However, there is data involving gene bodies and CpG “shores” (regions situated near CpG islands—upstream or downstream) that despite the abnormal methylated status within cancer, the downstream effect does not consist of silencing the transcriptional and translational processes, a fact that suggests that the spatial context (localization of the CpG sites within the genome) is actually a crucial element in the establishment of gene silencing [20].

Although the hypermethylation of CpG islands is the most common subject in cancer epigenetics, the actual malignant epigenetic profile is associated with a global hypomethylation trend. Also, restrictive data has been presented about the status of the genes responsible for the methylation process—DNA methyltransferases (DNMTs)—and also of those responsible for the expression of methyl-binding proteins accountable for recruiting histone-modifying enzymes [21]. Mutations in these genes can heavily impact the epigenetic signature [22] and implicitly lead to the induction of different diseases. As said, extensive analysis regarding the integrity of the reminded genes in cancer samples is still at the beginning but with the potential of revealing new layers of regulation in terms of cancer epigenetics. One such study conducted by Ley et al. found that *DNMT3A* is mutated in approximately 25% of patients diagnosed with acute myeloid leukemia (AML); those authors also associated the mutations with an impact on the prognosis of such individuals [23].

## 4. Histone Modifications

Histone proteins are predisposed to different modifications comprising ubiquitylation, sumoylation, methylation, acetylation, and also phosphorylation. These modifications take place preferentially at the N-terminal tails, affecting gene transcription and vital signaling pathways [24]. As opposed to DNA methylation, histone covalent modifications can also promote transcription and not only silence the expression of specific genes. As a practical example, lysine acetylation is majorly associated with the accessibility of transcription machinery to the chromatin and implicit transcription promotion, where lysine methylation does not follow necessarily the same pattern. Depending on the specific localization of methylation, the epigenetic modifications can be correlated with transcription activation or repression. More specifically, methylation of H3 lysine 9 (H3K9), H3 lysine 27 (H3K27), and H4 lysine 20 (H4K20) is associated with inhibitory mechanisms, where methylation of histone H3 lysine 4 (H3K4) and H3 lysine 36 are conducted towards activation of chromatin transcription [9,25]. The landscape of histone modification is different depending on the cellular context and these differences contribute to specific cell behaviors, including cancer cells that lose the homeostatic epigenetic pattern. This pattern is coordinated through the activity of enzymes that are able to add or remove methyl and acetyl groups and also functionally interact in order to establish the specific epigenetic profile [9,26,27]. Modifications in the structure and activity of these enzymes were previously associated with cancer susceptibility and also development. For example, p300 HATs (histone acetyltransferases) were found as being mutated in gastrointestinal cancers where the counterpart molecules, HDACs (histone deacetylases), were less frequently encountered as being deregulated. For the case of methyltransferases, it was assessed that mice lacking the Suv39 family of enzymes responsible for the methylation of H3K9 are predisposed to malignant pathologies, especially B cell lymphomas. Aurora kinases, enzymes with histone phosphorylation capacity, have also been correlated with cancer mechanisms [28]. Oral cancer is characterized by increased histone acetylation where experimental impairment of p300 acetyltransferase significantly decreased the tumor parameters in mice models [29].

## 5. Nucleosome Positioning and Histone Variants

Nucleosome positioning and also histone variants are framed in the non-covalent modifications category, but also with important roles regarding the establishment of the cell-specific epigenome. The position of nucleosomes is especially important when looking at the accessibility of transcription factors to specific regions within the chromatin structure, a process that has direct effects on the inhibition or activation of gene expression [30]. Genomic mapping of these positions showed that the pattern is strictly controlled around gene promoters where the actual gene sequence is characterized by a more random distribution of nucleosomes. There are also nucleosome-free regions (NFR) that are considered to provide the “space” for the transcription machinery to self-assemble and also to disconnect from the gene. Modifications in nucleosome architecture, together with other regulatory epigenetic mechanisms, can lead to transcription impairment or stimulation where the addition of an NFR or the loss of another can drastically modify the cell phenotype [31,32,33,34,35].

Histone variants—for example, H3.3 and H2A.Z—are usually incorporated throughout the cell cycle and further influence the expression of genes, mainly through activation due to assimilation within adjacent promoter areas and also protection of DNA methylation [36,37].

## 6. Non-Coding RNAs—miRNAs

miRNAs belong to the non-coding group of sequences and are able to modulate the expression of specific genes based on complementarity rules. These small molecules of approximately 22 nucleotides in length bind to the 3′UTR of the mRNA and block the translation of the target gene or, even more, can induce complete degradation of the transcript [38,39,40]. Therefore, miRNAs have emerged as key molecules within the regulation of signaling pathways, being able to modulate the expression of their target gene and implicitly maintain the normal state of the organism [41,42,43]. In terms of malignant pathologies, there are a great number of studies that have demonstrated that miRNAs are aberrantly expressed in cancer cells, a fact that contributes to the maintenance and also the development of the pathology. More specifically, the pattern consists of downregulated tumor suppressor miRNAs that in normal states inhibit the activity of oncogenic genes, and upregulated tumor promoting miRNAs that are able to impair the translation of the tumor suppressor ones. Subsequent to these discoveries, researchers have implemented experimental protocols where the pathological miRNA pattern is modulated for therapeutic purposes through administration of exogenous sequences (miRNA mimics or inhibitors) or for implementation of novel diagnosis and prognosis tools (considering the fact that miRNAs are differentially expressed in tumor or fluid samples vs. control ones) [44,45].

However, how is the global expression of the miRNA panel (miRNome) maintained during the normal scenarios and how is it lost in pathological ones? Studies have shown that miRNAs are also subjected to epigenetic regulation, such as protein-coding genes, and can also target specific effectors within the epigenetic machinery, such as enzymes for DNA methylation (DNMT3A and DNMT3B) and also those for histone modification (EZH2) [46,47,48,49,50,51]. Therefore, these regulatory pathways once again demonstrate the complexity of the epigenetic landscape and also the elaborated pathways involved in cancer induction, maintenance, and development.

## 7. Epigenetic Landscape in Oral Cancer

**Figure 2:**
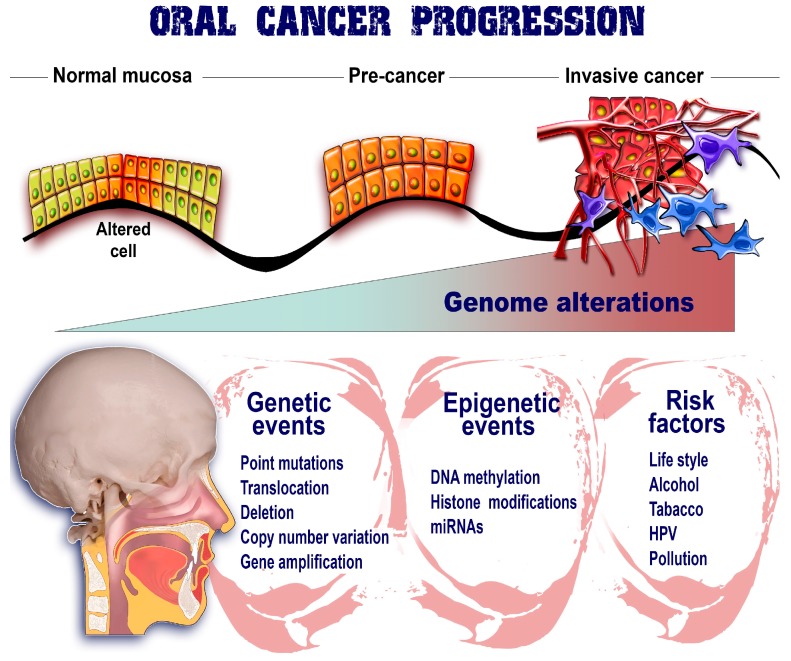
Epigenetic marks upon oral cancer. The evolution of oral cancer from a few altered cells to invasive phenotypes able to metastasize and populate secondary sites is the consequence of numerous factors that are interplaying their roles. Therefore, genetic events together with risk factors are combined with epigenetic mechanisms in order to ensure the proper environment for malignant development. All these factors gradually contribute to the organization of an unstable genome and also implicitly to the promotion of cancer advancement.

As previously discussed, the epigenetic marks represent an important layer of regulation within normal cells that maintain the homeostatic state and also the specific phenotype. An emerging number of studies have demonstrated that this strict regulatory mechanism is lost in cancer cells with consequences that favor the malignant niche and the advancement toward metastatic spread. Studies of these mechanisms have provided new etiologic perspectives for oral cancer genesis with an increased focus on DNA methylation, histone modifications, and miRNA regulation (Figure 2) [52,53,54].

## 8. DNA Methylation in Oral Cancer

A comparison of normal versus tumor samples of oral cancer has demonstrated that there are significant differences between the epigenetic signatures, where the tumor samples exhibit increased genome-wide hypomethylation and also hypermethylation of promoter regions [52,55]. Hypomethylation can result in increased chromosome instability due to the release of repetitive elements within the genome and also possible activation of silenced proto-oncogenes by the removal of promoter hypermethylation. The opposite mechanism, hypermethylation, is also present in oral cancer where CpG sites within the promoter sequence of tumor suppressor genes are methylated, a fact that impedes the access of transcription factors to these segments and, implicitly, the expression of specific genes.

For oral cancer, certain risk factors such as tobacco and alcohol consumption and also chronic inflammation of the oral mucosa have been linked to dysregulations in the epigenetic pattern regarding the methylation status. For example, smokers have been associated with an increased global hypomethylation [56,57] where, on the other hand, alcohol consumers are characterized by CpG hypermethylation according to clinical studies effectuated on patient samples and also in murine models for oral malignancies. Furthermore, analysis of specific gene hypermethylation in 47 OSCC patients (*p16*, *DAPK*, *RASSF1A*, *APC*, *WIF1*, *RUNX3*, *E-cad*, *MGMT*, and *hMLH1*) revealed that the epigenetic status can be used as a prognosis tool, where DAPK promoter hypermethylation by its own can function as an independent parameter for overall survival (HR = 4.105) [58].

Much attention has been granted to epigenetic modifications of specific genes in order to unravel the molecular mechanism behind their behavior in oral cancer and also to find therapeutic alternatives to restore the normal epigenetic mechanisms and inhibit cancer development.

***APC* (adenomatous polyposis coli)** is a tumor suppressor gene that normally is translated into a multi-domain protein able to bind different molecules including β-catenin, which functions in adherence junctions and Wnt signaling. Mutations (loss) in (of) APC have been linked to the inability to bind β-catenin, which further accumulates in the nucleus and triggers the canonical Wnt signaling [59,60,61] pathway that is essential for tumorigenesis (cell proliferation and differentiation). The mutational profile of APC was also investigated in oral cancer using basic research protocols. It was found that this gene is mutated in the SAS oral cancer cell line but with no amino acid changes. Moreover, no changes were observed in the tumor samples from patients. However, in cell lines (75%) as well as clinical specimens (90%) an increased accumulation of β-catenin was found [62]. Even if this scenario seems like a disruption from the previous association between WT APC (wild-type APC) and β-catenin in oral cancer, the further evidence has demonstrated that in fact the promoter of the APC gene is increasingly methylated in OSCC patient tissue samples and also in cell lines compared to controls [63,64,65,66]. This could explain the accumulation of β-catenin in oral cancer even in the absence of a functional mutation in the *APC* gene (considering the fact that promoter methylation concludes with silencing of gene expression).

**Survivin** plays a major role in oral cancer and also in other types of malignancies by inhibiting the apoptotic mechanisms and also regulating cell cycle, favoring the overcoming of checkpoints. The expression of the gene has been found to be increased in different tumor samples, including the ones collected from oral cancer patients, and was also correlated with clinicopathological features; moreover, this gene is usually not expressed in normal tissue [67,68]. Overexpression of survivin in basic cancer research has been previously linked with the presence of several single nucleotide polymorphisms (SNPs) [69]. However, there is evidence that in oral malignancies this gene is actually hypomethylated [52,70,71], contrary to normal samples where hypermethylation prevents the increased expression.

**E-cadherin** is synthesized from the *CDH1* gene and is responsible for keeping the adhesion between cells intact. The loss of *CDH1* expression is frequent in numerous types of cancers, an event that facilitates the epithelial to mesenchymal transition (EMT) and implicitly the colonization of secondary sites (metastasis) [72]. *CDH1* silencing has been observed in OSCC and was also correlated with a highly aggressive pattern and poor prognosis in the clinical environment (OSCC patients). One of the proposed mechanisms for this downregulated pattern of expression consists of aberrant epigenetic regulation through increased hypermethylation [73,74,75,76,77]. However, the results are quite inconsistent, with values ranging from 7% to 46%; it was previously proposed in the literature that there is the need for standardization of the IHC-based methods and further assessment of the epigenetic-related modifications of *CDH1* [78]. Considering the strong impact of environmental risk factors on the epigenetic pattern and also the heterogeneity of these outside modulators and the phenotypic differences among different individuals, it is possible that these results will keep rolling in an unstable manner. A possible approach could be the development of a large multi-centric study for the evaluation of the epigenetic pattern in oral cancer patients where the outside values could be significantly attenuated.

***PTEN* (phosphatase and tensin homolog deleted on chromosome 10)** has been called the “new guardian of the genome,” playing a major role in the suppression of cell survival and proliferation but also differentiation, apoptosis, and invasion, ranking in second place regarding the frequency of mutations in cancer (after TP53) [79]. Hypermethylation of promoter regions has been linked to different types of malignancies (observations made in both clinical samples and cancer-specific cell lines), including cervical [80], gastric [81], endometrial [82], and also non-small-cell lung cancer [83]. Downregulation of *PTEN* was also observed in oral cancer with a possible connection to epigenetic modification: hypermethylation. Kurasawa and colleagues are one of the groups that sustain this mechanism for *PTEN* regulation in OSCC where an increased number of patients exhibited hypermethylation patterns, a mechanism persistent also in 4 out of 6 cell lines, where the transcript levels were found as significantly downregulated but with no associated mutations [84]. The exact mechanisms behind PTEN expression in oral cancer are still incompletely deciphered, although the cancer inhibitory role of this sequence is well established in other cancers. Patients lacking this functional gene are characterized by more aggressive forms of malignancies and also a poor prognosis. In this sense there are some contradictory opinions regarding the *PTEN* gene in both clinical and basic research [85,86,87,88,89]; some groups support the downregulated pattern where others do not follow the same data.

There are also several other genes proposed as being epigenetically regulated and direct participants in oral cancer progression through their aberrant level of expression in clinical specimens and different oral cancer cell lines. Table 1 presents a comprehensive list of these genes together with their role in carcinogenesis.

## 9. Histone Modifications in Oral Cancer

The process of aberrant histone modification in oral cancer is not as extensively studied as DNA hypermethylation/hypomethylation, although these two mechanisms work in close connection. Mancuso et al. found that OSCC tissue differs from its normal counterparts through H3K4 histone methylation patterns, where H3K4me2 levels were increased and H3K4me3 were at lower levels in clinical samples. They also investigated the status of H3K4me1 but no important changes were observed. This differential epigenetic signature between tumor and normal samples sustains the idea of aberrant histone modifications in cancer that further contribute to the preservation of the malignant phenotype [109]. A more recent study evaluated the global histone modifications in 186 patients with OSCC and found several epigenetic alterations that were also associated with tumor status and stage and also invasion. More specifically, H3K4ac was decreased whereas H3K27me3 was at higher levels in OSCC clinical samples. These two markers are correlated with advanced forms of oral cancer and also cancer-specific survival (CSS) and disease-free survival (DFS). The authors also proposed this pathological pattern to be a valuable prognosis tool in OSCC [110]. Besides the actual histone modifications, there are also enzymes that are accountable for the post-transcriptional modifications, such as histone deacetylase (HDAC) that is responsible for the acetylation degree of the histone tails and is implicitly involved in numerous processes associated with cancer: apoptosis, differentiation, and growth arrest [111]. As a result, HDAC 6 was associated with increased expression levels in the majority of the malignant samples when compared to TAMs, an increase that was also proportional to the cancer stage (in OSCC-derived cell lines and also clinical specimens) [112]. CAF-1 (chromatin assembly factor-1) is responsible for the organization of the nuclear chromatin status within DNA replication, being also found as deregulated in different oral cancer subtypes [113,114]. Moreover, the status of CAF-1 is considered as a possible prognosis marker in OSCC and is associated with specific clinical parameters: advanced stages and metastasis [113,114].

## 10. Epigenetic Alterations of miRNAs in Oral Cancer

Modification of the miRNAs profile represents a crucial event for cancer induction, development, and also invasion and metastasis, where the altered expression of these sequences is associated with malignant signaling pathways. Sethi et al. have comprehensively presented the most important miRNAs involved in head and neck cancers [115] together with their possible translated clinical role; however, in terms of epigenetic modulation of miRNAs, the data are much more restrictive for oral cancers. The miR-34 family is one of the best-studied groups with tumor suppressor functions in numerous types of malignancies, including oral cancer. Kozaki and colleagues found that miR-34a together with three other miRNAs, miR-137, miR-193a, and miR-203, are characterized by a constant downregulated expression in OSCC cell lines (RT7 as a control). Considering the fact that all of the four miRNAs are located near CpG islands, one of the hypotheses for the impaired expression involves the hypermethylation of the promoter regions. Treatment of the cells with 5-aza-2′-deoxycytidine, a potent demethylation agent, restored the expression of the four miRNAs, sustaining the epigenetic regulation of the reminded sequences. Moreover, ectopic expression of miR-137 or miR-193a in oral cancer basic research significantly improved cell growth, enforcing their tumor suppressor role [115]. Epigenetic modifications are also responsible for the cell-specific expression within different cellular entities. miR-200 s/miR-205 that are expressed in oral cancer compared with normal samples are actually inhibited in CD44^high^ oral CSCs (cancer stem cells) due to the lack of DNA hypermethylation [116]. These data from multiple sample types in OSCC reinforce the complexity of epigenetic and genetic regulations within cancer and cell microenvironments, regulations that are aberrantly driven towards the maintenance of the malignant invasion. The most important dysregulated miRNAs in oral cancer are presented in Table 2.

Another possible field of study could be represented by the miRNAs that possibly target the enzymes involved in establishing the epigenetic landscape. These sequences could pathologically modify the expression of the epigenetic-related enzymes and lead to the establishment of differential epigenetic profiles. Even so, this idea of study is still limited within the scientific literature, especially in the context of oral cancer.

## 11. Epigenetic Therapies in Oral Cancer

The reversible character of epigenetic modifications is currently explored in preclinical research for therapeutic purposes in different types of malignancies, including oral cancer. Zebularine is an inhibitor of DNA methyltransferase, where treatment of HSC-3 cell line (OSCC model) resulted in impaired cell growth and also a decreased population of cells situated in the G2/M cell cycle phase [128]. Even so, the untargeted character of zebularine remains a constant problem for the future implementation of this type of agent in the clinic. Zebularine combined with cisplatin promoted cell death via an apoptotic mechanism while the combination of the same compound with 5-fluorouracil minimized the action of the chemotherapeutic agent [129]. This type of strategy has been clinically tested in patients with head and neck cancer where cisplatin was combined with azacitidine (hypomethylation activity). Both of the two studies involving these types of therapeutic formulations have been terminated with no clear results (accrual problems) (ClinicalTrials.gov Identifier: NCT00901537 and NCT00443261). Natural agents are also being explored for potential epigenetic modifying abilities; green tea administrated in in vitro models of oral cancer revealed inhibitory effects that were associated with a reversal of *RECK* gene hypermethylation and increased expression [130].

Histone deacetylase inhibitors are gaining momentum for oral cancer treatment with the purpose of promoting the activity of tumor suppressor genes by suppressing the acetylation process and preserving the loose structure of chromatin. The most promising results were shown in the case of combined strategies where histone deacetylase inhibitors were administrated with chemotherapeutic agents in preclinical models in order to synergistically decrease the malignant development (e.g., cisplatin combined with MS-275) [131]. Other similar agents that were tested for histone epigenetic landscape modulation consist of trichostatin A, butyric acid derivatives, and romidepsin [131].

miRNAs also have an increased potential in terms of oral cancer therapeutics for the modification of the pathological epigenetic profile. As in the case of other types of malignancies, the non-coding profile of oral cancer is also altered, an event that contributes to the maintenance of the cancer hallmarks [51,132,133]. Even if the “classical” approach consists of introducing exogenous tumor suppressor sequences for their reinforced expression (and also inhibitor sequences for oncogenic miRNAs), some of the downregulated sequences could also be restored at their basal level with epigenetic modifiers. Saito et al. applied this type of strategy by administrating epigenetic drugs (4-phenylbutyric acid and 5-aza-2′-deoxycytidine) in human cancer cell lines for the promotion of miR-127 levels and downstream inhibition of *BCL6* (B-cell lymphoma 6) [134]. Even if miR-127-specific therapy is not valid for oral cancer (found as overexpressed OSCC tissue samples compared to control [116]), similar specific tumor suppressor miRNA could be restored to their homeostatic level through this strategy. For example, miR-200s and miR-205 were associated with decreased expression in oral malignant tissue versus healthy controls where their activation corresponded to low CpG methylation [116]. Another perspective could be represented by the restoration of miRNAs that normally inhibit the aberrant activity of enzymes such as DNA methyltransferase, as in the case of zebularine administration. However, the constant issue remains the nonspecificity of these epigenetic modifier drugs where healthy cells could also be affected or, moreover, oncogenic genes previously suppressed even in cancer cells could be activated (an event that could decrease the efficiency of the experimentally activated tumor suppressor genes).

## 12. Conclusions

Epigenetic mechanisms have gained their role in the cancer hallmarks; it is now clear that these types of alterations are also part of the heterogeneous cancer signaling. One steady advantage is the reversible feature of epigenetics, where the aberrant signature can be modified through the administration of exogenous inhibitors of DNA methyltransferase or histone deacetylase. The idea of combining the standard therapy with new epigenetic modulatory agents could improve significantly the clinical outcome considering that, for example, genes related to chemoresistance have been found as hypermethylated [135]. In this way, the efficiency of the classical treatment could be significantly improved. These preliminary results in oral and also other types of cancer are promising where most probably it is just a matter of time before these types of therapeutic agents (epigenetic modifiers) will gather more attention in clinical practice.

## Figures and Tables

**Table 1:** Epigenetically regulated genes with implications in oral cancer progression.

Gene	Mechanism	Locus	Epigenetic Modification	Ref
*CDKN2A/p16*	Cell cycle, senescence	9p21	Hypermethylation	[73,74,90,91,92]
*CDH1/E-cadherin*	EMT, adhesion	16q22.1	Hypermethylation *	[73,74,75,77]
*PTEN*	Differentiation, survival, proliferation, invasion, apoptosis	10q23.3	Hypermethylation *	[84,89]
*DAPK1*	Apoptosis	9q34.1	Hypermethylation	[93,94,95]
*MGMT*	DNA repair	10q26	Hypermethylation	[90,92,94,96]
*RARB2*	Cell proliferation	3p24	Hypermethylation	[97,98]
*RASSF1/2*	Cell cycle, apoptosis, and microtubule formation	3p21.3/20p13	Hypermethylation	[99,100]
*APC*	Cell proliferation	5q22.2	Hypermethylation	[64,65]
*Survivin*	Cell proliferation and apoptosis	17q25	Hypomethylation	[70,71,101]
*MLH1*	DNA repair	3p22.2	Hypermethylation	[73,102,103]
*p14(ARF)*	Cell proliferation, division, angiogenesis	9p21	Hypermethylation	[104,105,106]
*p15INK4B*	Cell cycle	9p21.3	Hypermethylation	[104,107]
*p16INK4A*	Cell cycle, senescence	9p21	Hypermethylation	[73,74,90,91,92]
*RARβ*	Cell growth and differentiation	3p24	Hypermethylation *	[97,108]

* Controversial results or unclear role in oral cancer.

**Table 2:** Most significant dysregulated miRNAs in oral cancer.

miRNA	Type of Malignancy	Possible Clinical Utility	Expression	Reference
miR-21	Head and neck squamous cell carcinoma, Oral Squamous cell carcinoma	Diagnosis/prognosis utility (detected also in plasma)	Upregulated	[117,118]
miR-375	Oral squamous cell carcinoma	Associated with clinical parameters	Downregulated	[119]
miR-31	Oral carcinoma	Non-invasive and early diagnosis tool (saliva) and also prognosis marker	Upregulated	[120]
miR-7	Oral squamous cell carcinoma		Upregulated	[118]
miR-27b	Oral squamous cell carcinoma	Saliva biomarker for OSCC	Upregulated	[121]
miR-125b	Oral squamous cell carcinoma	Therapeutic target	Downregulated	[122]
miR-155	Oral squamous cell carcinoma	Prognosis value	Upregulated	[123,124]
miR-181	Oral squamous cell carcinoma	Lymph node metastasis marker	Upregulated	[125]
miR-211	Oral carcinoma	Poor prognosis marker	Upregulated	[126]
Additional upregulated miRNAs	miR-9*, miR-424, miR-7–1*, miR-15b, miR-9, miR-155, and miR-196a, miR-24, miR-18a, miR-221, miR-16, let-7b,			[118,121,127]
Additional downregulated miRNAs	miR-486-5p, miR-136, miR-147, miR-1250, miR-148a, miR-632, miR-646, miR-668, miR-877, miR-503, miR-220a, miR-323-5p, miR-223, miR-29a			[118,121,127]
